# Isolation, Identification, and Characterization of Antibiotic-Producing Bacteria from the Phyllosphere of *Zea mays*

**DOI:** 10.17912/micropub.biology.001777

**Published:** 2025-08-08

**Authors:** Katie Christensen, Hanna Lefevers, Bashir Akhlaq Akhoon, Kendall R. Corbin

**Affiliations:** 1 Department of Horticulture, Martin-Gatton College of Agriculture, Food and Environment, University of Kentucky, Lexington, KY, USA

## Abstract

Plants are a largely untapped source of antibiotic-producing microorganisms. In this study, 237 bacteria were isolated from the leaf surface of field-grown *Zea mays* . Screening 49 Gammaproteobacteria isolates for antibiotic activity identified eighteen isolates of interest. These findings underscore the plant phyllosphere as a valuable reservoir for discovering novel antibiotics.

**Figure 1. Overview of maize-associated bacterial isolates with antibiotic-producing potential and their phylogenetic relationships:**
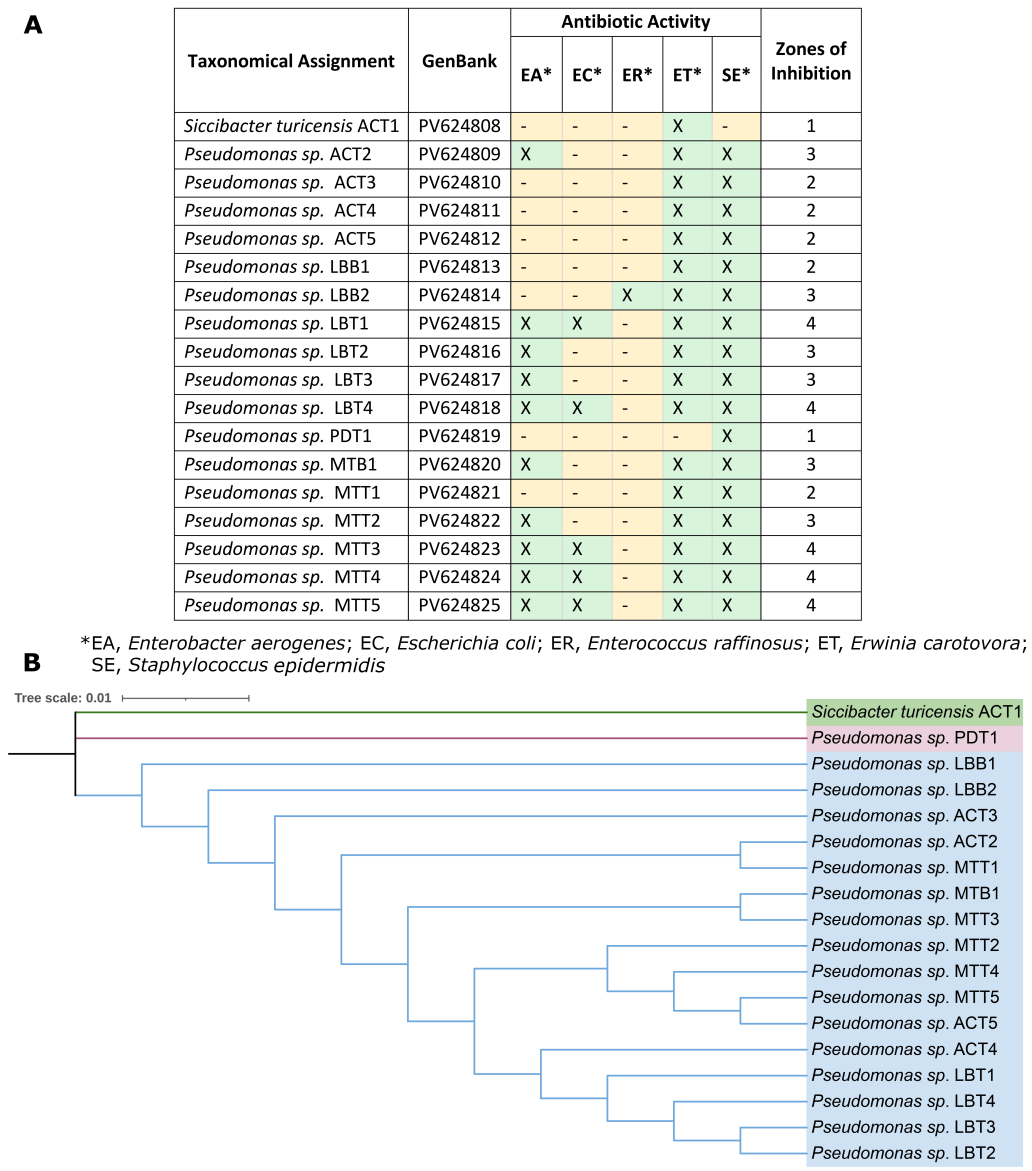
(A) Taxonomical assignment, GenBank accession numbers and antibiotic activity results for bacterial isolates collected from maize leaves. (B) The maximum likelihood phylogenetic tree inferred from 16S rRNA gene sequences shows the relationships among *Pseudomonas* isolates and the outgroup *Siccibacter turicensis* (ACT1). The outgroup forms a distinct branch, whereas all *Pseudomonas* isolates cluster within a well-supported monophyletic clade.

## Description

Maize ( *Zea mays* L.) is a globally significant cereal crop, serving as a vital source of food, livestock feed, and a resource for bioenergy production. The United States, Brazil, and China rank among the top producers of maize worldwide (USDA, 2020), highlighting its broad agricultural and economic importance. Beyond its value as a crop, maize also supports a complex microbial ecosystem on its aerial surfaces, collectively referred to as the phyllosphere. The phyllosphere harbors a wide range of microorganisms, including bacteria, fungi, and other microorganisms (Xiang et al. 2024; Sarver et al. 2025). Among these, bacteria dominate and contribute significantly to plant health and ecosystem functioning. Studies have consistently identified *Proteobacteria* and *Actinobacteria* as the most prevalent bacterial phyla inhabiting maize leaves (Kong et al. 2020). Within these phyla, *Pseudomonadaceae* , *Erwiniaceae* , and *Alcaligenaceae* have emerged as key members of the maize phyllosphere microbiome (Wu et al. 2023). Genetic variation among maize cultivars plays a crucial role in shaping the composition and functional attributes of these microbial communities (Kong et al. 2020). Additionally, the physical and biochemical traits of maize leaves—such as leaf surface area and chlorophyll content—have been positively linked to bacterial abundance (Tang et al. 2023). Beneficial microorganisms residing on the surface of plant leaves play a key role in suppressing plant pathogens. One of the primary mechanisms by which these microorganisms contribute to plant health is through the production of antibiotics (Gerardin et al. 2016), which can directly inhibit the growth and spread of harmful organisms. In addition to antibiotic synthesis, these phyllosphere-associated bacteria also compete with pathogens for space and nutrients and can stimulate the plant’s own defense responses (Du et al. 2024; Zhu et al. 2022). Together, these interactions create a protective microbial barrier that limits disease development and enhances overall plant resilience.

In this study, bacteria were isolated from the phyllosphere (leaves) of maize. Using Sanger sequencing, 237 isolates were identified. The antibiotic potential of 49 isolates from the class Gammaproteobacteria (4 *Actinobacter* , 2 *Enterobacter* , 7 *Pantoea* , 34 *Pseudomonas* , 1 *Siccibacter* , and 1 *Stenotrophomonas* ) was assessed against safe relatives of ESKAPE pathogens ( *Enterobacter aerogenes, Escherichia coli, Enterococcus raffinosus, Erwinia carotovora, and Staphylococcus epidermidis* ). Five isolates exhibited broad-spectrum antibacterial activity ( Figure 1A ), suggesting that plants are an understudied reservoir of antibiotic-producing bacteria.

Of the isolates tested, 37% (18 isolates: 17 *Pseudomonas* and 1 *Siccibacter* ) produced zones of inhibition (antibiotic activity) against at least one safe ESKAPE pathogen relative. Five isolates showed antibiotic producing potential ( Figure 1A ) against 4/5 of safe ESKAPE relatives. Based on 16S rRNA Sanger sequencing, these isolates were identified as *Pseudomonas* sp. and *Siccibacter turicensis* . A phylogenetic tree based on 16S rRNA gene sequences was constructed to elucidate evolutionary relationships among the bacterial isolates ( Figure 1B ). Sequence alignment, taxonomic classification, and tree inference were performed using the Alignment, Classification and Tree (ACT) Service provided by the SILVA database ( www.arb-silva.de/aligner ), employing RAxML (Randomized Axelerated Maximum Likelihood) with default parameters for maximum likelihood-based phylogenetic reconstruction.

The phylogenetic analysis revealed clear taxonomic structuring among the *Pseudomonas* isolates, with all species forming a well-supported monophyletic clade distinct from *Siccibacter turicensis* (ACT1), which was separated by a comparatively long branch. Within the *Pseudomonas* clade, *Pseudomonas sp. PDT1* diverged earliest, forming a basal lineage relative to the remaining isolates. *Pseudomonas sp. LBB1* and *LBB2* grouped closely together, indicating high genetic similarity and likely representing sister taxa. These were further related to a larger, deeply nested subclade that included *ACT3* , *ACT2* , *MTT1* , *MTT3* , *MTB1* , and multiple other isolates. Notably, several subgroups emerged within this cluster: one composed of *MTT1* and *ACT2* ; another joining *MTT3* with *MTB1* ; and a highly structured group comprising *ACT4* , the *LBT* series (LBT1–LBT4), and *MTT2–MTT5* along with *ACT5* . The internal topology within this major *Pseudomonas* clade suggests a pattern of fine-scale diversification, likely reflecting recent evolutionary divergence or adaptation to distinct ecological niches. The extremely short branch lengths within the *Pseudomonas* groups indicate high overall sequence similarity, consistent with close phylogenetic relationships among strains, possibly within the same species complex. The 16S rRNA gene sequences generated in this study have been deposited in GenBank and are publicly available under the following accession numbers: *Siccibacter turicensis* – PV624808 ; *Pseudomonas* sp. – PV624809 , PV624810 , PV624811 , PV624812 , PV624813 , PV624814 , PV624815 , PV624816 , PV624817 , PV624818 , PV624819 , PV624820 , PV624821 , PV624822 , PV624823 , PV624824 , and PV624825 . All sequences were obtained by Sanger sequencing. The version described here is the first release and is accessible via the NCBI Nucleotide database .

This study highlights the untapped potential of plant-associated microbes as sources of novel antibiotics. The maize-phyllosphere biobank developed here provides a valuable resource to advance research on plant–microbe interactions and antimicrobial discovery.

## Methods

Sterile flock swabs dipped in a sterile wash solution were used to sample microorganisms from the upper and lower surfaces of field-grown maize plants (Smets et al. 2023). Plants (G14R38-GT) were grown at the University of Kentucky (UK) North Farm in Lexington, Kentucky for 5 weeks. Serial dilutions of the swabs were prepared and plated on four types of medium- All Culture agar, Luria Broth agar, Potato Dextrose agar, and Tryptic Soy agar. The plates were incubated at 27°C. Individual colonies were isolated through successive streaking, preserved in glycerol, and stored at -80°C. ZymoBIOMICS™ 96 MagBead DNA Kit was used for DNA extraction with the v14 library prep chemistry kit from ONT. For taxonomical identification, the 16s rRNA gene was amplified and sequenced using the Tiny Earth colony-bead PCR method (Hernandez et al. 2020) and Sanger sequencing at the UK Genomics Core Laboratory. In brief, the 16S rRNA 27F primer, the 1492R primer, a colony and water were added to Cytiva PuReTaq Ready-To-Go™ PCR beads (25µL). The PCR settings were (i) 1 cycle of 94°C for 10 min, (ii) 30 cycles of 94°C for 30 s, 58°C for 30 s, and 72°C for 1 min 50 s, and (iii) 1 cycle of 72°C for 10 min. Three primers were used for sequencing (27F-AGAGTTTGATCMTGGCTCAG; 515F-GTGCCAGCMGCCGCGGTAA; and 1492R-CGGTTACCTTGTTACGACTT). Sequences were trimmed, aligned, and combined using Geneious Prime’s *de novo* assembly. Isolates were identified using the NCBI Basic Local Alignment Search Tool (BLAST) and the Core Nucleotide database. In total, 49 isolates were classified as Gammaproteobacteria (4 *Actinobacter* , 2 *Enterobacter* , 7 *Pantoea* , 34 *Pseudomonas* , 1 *Siccibacter,* and 1 *Stenotrophomonas* ). To investigate the antibiotic producing potential of these isolates, safe relatives of the ESKAPE pathogens were used ( *Enterobacter aerogenes* ATCC51697 *, Escherichia coli* ATCC25922 *, Enterococcus raffinosus* ATCC49464 *, Erwinia carotovora* ATCC25270 *, and Staphylococcus epidermidis* ATCC14990). Isolates were grown with the safe ESKAPE relatives using the patch method. After 3 days (27°C), the plates were observed for zones of inhibition (Hernandez et al. 2020).
